# ‘One of the Best Fathers until He Went Out of His Mind’: Paternal Child-Murder, 1864–1900

**DOI:** 10.1080/13555502.2012.751045

**Published:** 2013-02-26

**Authors:** Jade Shepherd

**Affiliations:** ^a^ Queen Mary, University of London

**Keywords:** Asylums, Broadmoor, Crime, Fatherhood, Infanticide, Insanity, Masculinity, Murder, Trials

## Abstract

Current scholarship suggests that when a mother murdered her child in Victorian England she was treated sympathetically by the press and in the courtroom. It is argued that because the crime was considered antithetical to womanhood it was viewed as an indication of insanity. This article examines newspaper reports, trial transcripts, medical literature and popular works on fatherhood, in order to explore the cases of sixty men committed to Broadmoor Criminal Lunatic Asylum between 1864 and 1900 for the murder of their children. It questions two assumptions of the literature on infanticide: first, the idea that it was only women who were thought to be going against nature if they killed their child; and second, that it was only women who regularly successfully pleaded insanity in such cases. The Broadmoor case studies not only demonstrate Victorian attitudes towards paternal child-murder but also provide valuable material illustrating affectionate models of Victorian fatherhood. In trial and press reports detailing the crimes it is clear that fathers were expected, and expected themselves, to be temperate, provide for, and protect their children.

In March 1873, George Wilson murdered his ten-year-old son, Thomas. According to a medical report, Wilson ‘became disturbed and distracted by domestic […] troubles and finally gave way to intense melancholy, accompanied by delusions and strong religious fancies’, and while his mind was under these ‘insane influences’ he murdered his child.^1^ At the Old Bailey on 24 November 1873, witnesses testified to the distinct change in Wilson's character as a result of his insanity. His neighbour, Mr Oxley, told the court that Wilson would not have allowed ‘a hair on […] [his children's] heads to be injured’, and Wilson's eldest son William, when asked ‘Has he always been a kind father?’ replied ‘Oh, yes: he has been one of the best of fathers until he went out of his mind’.^2^ The jury found Wilson ‘to be of unsound mind and incapable of pleading to the indictment’ and he was committed to Broadmoor Criminal Lunatic Asylum.^3^
 1. Berkshire Record Office (BRO), Medical Report, D/H14/D2/2/1/811.
 2. ‘Dreadful Murder in London’, *Birmingham Daily Post*, 10 November 1873.
 3. *Old Bailey Proceedings Online* (www.oldbaileyonline.org, version 6.0, 20 June 2011), November 1873, trial of GEORGE WILSON (54) (t18731124-32).


During the 1820s physicians refined and developed the term infanticide as a symptom of puerperal insanity.^4^ Since Victorian psychiatrists (alienists) cast infanticide as maternal, scholars have tended to focus on infanticidal women and questions surrounding illegitimacy, poverty and puerperal insanity. Partly because of contemporary beliefs about womanhood, infanticidal women were usually considered insane.^5^ Cases of paternal child-murderers in nineteenth-century England are less frequently subject to historical research. Melissa Valiska Gregory has studied male infanticide in the early-nineteenth century and in *Child Murder and British Culture, 1720*–*1900*, Josephine McDonagh considers literary representations of male child-murderers in the eighteenth and nineteenth centuries. Both scholars suggest that murderous fathers were treated unsympathetically, as savage tyrants.^6^ Jill Newton Ainsley and Ginger Frost both argue that mothers who murdered their children were shown leniency in comparison to fathers who were more likely to be punished.^7^
 4. Hilary Marland, ‘Getting Away With Murder? Puerperal Insanity, Infanticide and the Defence Plea’, in *Infanticide: Historical Perspectives on Child Murder and Concealment, 1550*–*2000*, ed. by Mark Jackson (Aldershot: Ashgate Publishing, 2002), pp. 168–92 (p. 173).
 5. For example, Meg Arnot, ‘Understanding Women Committing Newborn Child Murder in England’, in *Everyday Violence in Britain, 1850*–*1950: Gender & Class*, ed. by Shani D'Cruze (Harlow and New York: Longman, 2000), pp. 55–69; Hilary Marland, *Dangerous Motherhood: Insanity and Childbirth in Victorian Britain* (Basingstoke: Palgrave Macmillan, 2004).
 6. Josephine McDonagh, *Child Murder and British Culture, 1720*–*1900* (Cambridge: Cambridge University Press, 2003), pp. 35–44; 178–183; Melissa Valiska Gregory, ‘“Most Revolting Murder by a Father”: The Violent Rhetoric of Paternal Child-Murder in *The Times* (London), 1826–1849’, in *Writing British Infanticide: Child-Murder, Gender, and Print, 1722*–*1859*, ed. by Jennifer Thorn (London: Associated University Press, 2003), pp. 70–90.
 7. Jill Newton Ainsley, ‘“Some Mysterious Agency”: Women, Violent Crime, and the Insanity Acquittal in the Victorian Courtroom’, *Canadian Journal of History*, 35.1 (2000), 37–55 (p. 45); Ginger Frost, ‘“I Am Master Here”: Illegitimacy, Masculinity and Violence in Victorian England’, in *The Politics of Domestic Authority in Britain Since 1800*, ed. by Lucy Delap, Ben Griffin and Abigail Wills (Baskingstoke: Palgrave Macmillan, 2009), pp. 27–42. Carolyn Conley has argued that gender did not determine the verdict in infanticide trials. See, *Certain Other Countries: Homicide, Gender and National Identity in Late-Nineteenth Century England, Ireland, Scotland and Wales* (Columbus, OH: Ohio State University Press, 2007), p. 22.


Analysis of the case files of 60 paternal child-murderers who were found insane and committed to Broadmoor between 1869 and 1900 suggests a different picture. An examination of trial records, medical reports, psychiatric treatises and newspaper articles, questions two previous assumptions of the literature on infanticide: first, that it was only women who were thought to be going against nature if they killed their child; and second, that only women regularly and successfully pleaded insanity in such cases.^8^ The release of the Broadmoor records for public viewing in November 2008 allowed the experience of its patients to be researched by historians for the first time. This article considers how paternal child-murderers were treated within the asylum and traces the experience of some patients from the time they committed child murder, until they were released from Broadmoor.^9^ An analysis of all of these sources not only demonstrates attitudes towards paternal infanticide, but also discloses a surprising and valuable source of material illustrating the kindness and affection of working-class fathers in the late nineteenth century. Analyses of nineteenth-century art, census documents, fiction, employment records, autobiographies, memoirs and legal cases have revealed a myriad of representations of fatherhood in recent years.^10^ This article enriches such research on nineteenth century fatherhood by showing that working-class fathers were expected, and expected themselves, to be hardworking and temperate, as well as to provide for and protect their children. 8. The cases analysed here are not representative of all of the men tried in England for murdering their child between 1864 and 1900, nor are they representative of all the men committed to Broadmoor for the crime. Exact numbers of paternal murders are difficult to ascertain because the crimes committed by patients were not always recorded in Broadmoor's admission registers. This article derives from a wider research project examining the crimes, trials and incarceration of men committed to Broadmoor between 1863 and 1900.
 9. For the discharge of infanticidal women from Broadmoor, see Jonathan Andrews, ‘The Boundaries of her Majesty's Pleasure: Discharging Child-Murderers from Broadmoor and Perth Criminal Lunatic Department *c.* 1860–1920’, in *Infanticide*, ed. by Mark Jackson, pp. 216–48.
10. See for example, *Gender and Fatherhood in the Nineteenth Century*, ed. by Trev Lynn Broughton and Helen Rogers (Basingstoke: Palgrave Macmillan, 2007).


## I. Charles Dickens and other child-killers

In 1841 Charles Dickens murdered his literary offspring little Nell Trent. In the days that followed the act, his real-life daughter, Mamie, later recalled:

like a father he mourned for his little girl – the child of his brain – and he writes: ‘I am, for the time, nearly dead with work and grief for the loss of my child’ […] ‘You can't imagine (gravely I write and speak) how exhausted I am to-day with yesterday's labors [sic]. I went to bed last night utterly dispirited and done up. All night I have been pursued by the child; and this morning I am unrefreshed and miserable. I do not know what to do with myself’.^11^
11. Mamie Dickens, *My Father As I Recall Him* (New York: Haskell House Publishers, 1974), p. 51.

In his day-to-day life, Dickens was, according to Mamie, an extremely tender, affectionate and loving father: ‘I can remember with us, his own children, how kind, considerate and patient he was’.^12^ Mid-to-late nineteenth-century discourse on fatherhood shows that attitudes towards fatherhood were changing; the kind and affectionate father epitomized by Dickens was, at least on paper, the expected norm.^13^ In his article ‘Fathers’, published in *All the Year Round* in 1865, Andrew Halliday observed, ‘The British father has undergone a great metamorphosis of late’. The modern father, he continued, ‘has relaxed his old severity aspect and become more human. […] Love and sympathy and intelligent communion have taken the place of a cold and senseless severity’.^14^ Thirty years later, in the advice manual *Happy Homes and How to Make Them* (1897), J. W. Kirton declared that it was a man's duty to:

rock a cradle, nurse a baby, [and] play with his children […]. It is a grand thing to have a romp with the children, and […] a man is not worthy to be a father who cannot now and then play with them, or take an interest in their sports and education.^15^
15. John William Kirton, *Happy Homes and How To Make Them, or, Counsels on Love, Courtship and Marriage* (London: John Kempster and Co, 1897), p. 97.

This was a popular message of marriage and courtship manuals while authors such as Anthony Trollope wrote of devoted and caring fathers in their fiction.^16^ A man was supposed to delight in his children and Charles Kingsley, following the birth of his first child, declared ‘my little baby, the next link in the golden chain of generations, begotten of our bliss’.^17^
12. Dickens, *My Father,* pp. 12–13; 16–17; 26.
13. For Victorian fatherhood see John Tosh, *A Man's Place: Masculinity and the Middle-Class Home in Victorian England* (New Haven, CT and London: Yale University Press, 2007).
14. Andrew Halliday, ‘Fathers’, *All the Year Round*, 14 (2 September 1865), 133–35 (p. 135).
16. Margaret Marwick, ‘Hands on Fatherhood in Trollope's Novels’, in *Gender and Fatherhood*, ed. by Broughton and Rogers, pp. 85–95.
17. Susan Chitty, *The Beast and the Monk: A Life of Charles Kingsley* (London: Hodder and Stoughton, 1974), p. 98. For Charles Kingsley and fatherhood, see Valerie Sanders, ‘“What Do You Want to Know About Next?” Charles Kingsley's Model of Educational Fatherhood’, in *Gender and Fatherhood*, ed. by Broughton and Rogers, pp. 55–67.


Research on fathers committed to Broadmoor for the murder of their children shows that it was not only in Dickens's life and work that a connection between infanticide and affection in the nineteenth century can be seen. The cases examined for this article demonstrate that prior to committing their crime, many working-class men revelled in their children; they nursed them, played with them, and were kind and affectionate towards them. At Richard Hammett's trial, his son told the court that he ‘was always very fond of us all—when we were ill he used to sit up with us at night and nurse us’; Joseph Wood had murdered his infant daughter without motive, ‘being a very affectionate father’; Frederick Crawley was ‘dotingly fond of the child’ he murdered; and at the trials of William Robinson and William Brown, it was said they were both ‘very fond’ of the children they murdered.^18^ They were all committed to Broadmoor.18. BRO, Schedule A, D/H14/D2/2/1479; Letter to Nicolson, D/H14/D2/2/1/1284/25; ‘Child Murder at Liverpool’, *Manchester Times*, 25 February 1871.


The defence counsels of paternal child-murderers generally argued that because defendants were affectionate and caring fathers they had no motive for committing the crime and therefore should be considered insane. Henry Seyman murdered his son Harry in 1869, and his defence argued:

Motive was of utmost importance, and that in the present case no motive had been shown. […] What! were they to believe that a kind father would at once throw off the bright instincts of his heart, and all the better feelings of nature, and become in a moment a cold blooded murderer?

The jury did not think so and found Seyman not guilty on the ground of insanity.^19^ In 1884 Alfred Bligh murdered his three children. Bligh's defence told the court they ‘could not conceive any case which was any more an obvious, more flat, more flagrant, contradiction of the natural tendencies of human nature than the act committed by that man’.^20^ Oonagh Walsh argues that female infanticide was considered antithetical to womanhood and thus ‘[i]nfanticide was treated sympathetically because it was so contrary to gendered expectations that it automatically implied an act of insanity’.^21^ A close study of the Broadmoor cases suggests that the same can be said of paternal child-murder: an act deemed so contrary to the expectations of fatherhood it was considered a contradiction of human nature. Thus, a more nuanced understanding of how the meanings of gender operated in child-murder trials is needed.19. ‘The Sheffield Child Murder’, *Sheffield and Rotherham Independent*, 3 April 1869, 3.
20. ‘The Kirkham Tragedy’, *Manchester Times*, 17 July 1886.
21. Oonagh Walsh, ‘Gender and Insanity in Nineteenth-Century Ireland’, in *Sex and Seclusion, Class and Custody: Perspectives on Gender and Class in the History of British and Irish Psychiatry*, ed. by Jonathan Andrews and Anne Digby (Amsterdam and New York: Rodopi Press, 2004), pp. 69–94 (p. 81).


Increasing interest in the study of children from the 1870s initiated a shift in the scientific community and men of science became more concerned with questions related to fatherhood and the connection between men and children.^22^ In his *The Emotions and The Will* (1875) Alexander Bain discussed the ‘Emotions of Parents’. He observed that not only were fathers sensitive and loving, but that ‘[t]he feeling of protectorship is cherished’ by them.^23^ As Megan Doolittle suggests, the protection of children was embedded in constructions of fatherhood and manliness. The cases from Broadmoor show that the delusions men suffered revolved around their fear of being unable to live up to the expectation that they would protect their child leading to the terrible paradox of killing a child out of a desire to protect.^24^ Edward Abbott, for example, killed his young daughter because he believed that his imminent murder would leave her vulnerable and Henry Garrod murdered his daughter because, having lost his money, he feared that she would grow up to lead ‘a bad life [and] be killed by Jack the Ripper’.^25^
22. Cynthia Nelson, *Invisible Men: Fatherhood in Victorian Periodicals, 1850–1900* (Athens, GA and London: University of Georgia Press, 1995), pp. 73–106; Sally Shuttleworth, *The Mind of The Child: Child Development in Literature, Science, and Medicine, 1840–1900* (Oxford: Oxford University Press, 2010), pp. 304–324.
23. Alexander Bain, *The Emotions and The Will*, 3rd edn (London: Longmans, Green and Co, 1875), p. 142.
24. Megan Doolittle, ‘Fatherhood, Belief and the Protection of Children in Nineteenth-Century English Families’, in *Gender and Fatherhood*, ed. by Broughton and Rogers, pp. 31–42.
25. BRO, Abbot's case file, D/H14/D2/2/1/799; Garrod's case file, D/H14/D2/2/1/1473.


It was the death of a child, as Leonore Davidoff and Catherine Hall suggest, that brought out paternal feelings most strongly.^26^ In Mary Elizabeth Braddon's novel *The Fatal Three* (1888), George Greswold is depicted as the ideal husband and father, but following the death of his daughter Lola from typhoid fever he is overwhelmed by grief and descends into madness.^27^ Representations of childhood death and male madness in real-life cases mirrored fictional tales. In 1875, James Senior murdered his ten-year-old daughter ‘in an attack of insanity’ driven by the ‘dread of losing [this] child who was […] seriously ill’ and in May 1890, Joseph Wood was tried for the murder of his three-week old daughter, Nelly, after he suffered an attack of insanity following the death of his son.^28^ At Wood's trial his mother, Ann, told the court that her grandson's illness had caused Wood to be ‘very weak and bad in his head’ and when he visited her ‘he sat down and cried’.^29^ The inability to save their child from death and disease led to feelings of hopelessness in both Senior and Wood and perhaps feelings of failure, just as in *The Fatal Three* where, as Valerie Pedler suggests, since ‘George has prided himself on his […] paternal devotion to Lola, her death seems to show a dismal proficiency in [this] respect’.^30^ A man's failure to protect his child not only led to mental demise, but emasculation.26. Leonore Davidoff and Catherine Hall, *Family Fortunes: Men and Women of the English Middle Class, 1780*–*1850* (London: Hutchinson, 1987), p. 30. See also, Julie-Marie Strange, *Death, Grief and Poverty in Britain, 1870*–*1914* (Cambridge: Cambridge University Press, 2005), pp. 261–262.
27. Mary Elizabeth Braddon, *The Fatal Three* (Leipzig: Bernhard Tauchnitz, 1888), pp. 50–51.
28. BRO, Senior case file, D/H14/D2/2/1/834.
29. *OBPO* (20 June 2011), May 1890, trial of JOSEPH WOOD (24) (t18900519-457). For male grief, Julie-Marie Strange, ‘“Speechless With Grief’’: Bereavement and the Working-Class Father, 1880–1914’, in *Gender and Fatherhood*, ed. by Broughton and Rogers, pp. 138–49.
30. Valerie Pedlar, *The Most Dreadful Visitation: Male Madness in Victorian Fiction* (Liverpool: Liverpool University Press, 2006), p. 128.


## II. Causes of crime and insanity: poverty and intemperance

Jill Newton Ainsley argues that the sympathy extended to mothers who killed their children because they did not want to subject them to a life of poverty was absent in cases of paternal infanticide.^31^ The Broadmoor records, however, indicate that poverty drove a number of fathers as well as mothers to murder their children: the fact they were sent to Broadmoor rather than to the gallows also indicates that a certain degree of sympathy was extended to them. John Tosh has highlighted the pressures Victorian fathers faced when it came to providing financially for their families.^32^ In his *Ascent of Man* (1899) Henry Drummond used evolutionary science to enforce the notion that it was a father's prerogative to provide for his children:

He is not only protector but food-provider. It is impossible to believe that in process of time the discharge of this office did not bring some faint satisfactions to himself, that the mere sight of offspring fed instead of famished did not give him certain pleasure. And though the pleasure at first may have been no more than the absence of the annoyance they caused by the clamorousness of their want, it became a stimulus to exertion, and led in the end to rudimentary forms of sympathy and self-denial.^33^
33. Henry Drummond, *The Ascent of Man* (London: Hodder and Stoughton, 1899), pp. 394–95. For Drummond on motherhood see Thomas Dixon, *The Invention of Altruism: Making Moral Meanings in Victorian Britain* (Oxford and New York: Oxford University Press, 2008), pp. 273–320.

A man's desire to provide for his family was viewed as innate, just like the need to protect them. Alienists, such as Charles Mercier, agreed:

The onset of poverty and adversity, which could easily be borne if the individual alone were concerned, may become a source of dangerous stress from the fact that it will involve offspring also; and anxiety as to their fate under such circumstances may be attended to by an amount of stress sufficient to produce insanity.^34^
34. Charles Mercier, *Sanity and Insanity* (London: Walter Scott, 1890), p. 275.

Henry Parker and Robert Hallowell both murdered their sons fearing they could not provide for them. The widower Alfred Bligh murdered his three children because he worried he would lose his job and subject them to a life of poverty.^35^ It was suggested at the inquest following Bligh's arrest that monetary problems and the impending affiliation of an illegitimate child weighed heavily on his mind.^36^ These issues seemingly exacerbated the mental deterioration of this struggling single father who, in his own words, had ‘been driven to do this’ for he could not ‘stand it any longer’.^37^ In *Crime and its Causes* (1891), criminologist and prison chaplain W. D. Morrison stated ‘it is probable’ that a man facing destitution ‘will commit crimes […] such as homicide or assault’.^38^ This is echoed in the case of Richard and Amy Oakes who, in 1890, poisoned their eight-year-old son Arthur. In an attempt to explain the act, Richard Oakes wrote a letter to his brother detailing his family's struggle to avoid destitution. In this ‘touching’ ‘letter of despair’, as it was referred to by the press, he described his struggle to find work and explained that for the six weeks previous to the crime he had ‘travelled from morning til night and not received one farthing’. He concluded, ‘[i]f that is not enough to drive you mad – wickedly mad – I don't know what is’.^39^ During the trial the defence argued that poverty had led the Oakes to become ‘partially, if not wholly, demented’ which had prompted the act. The Oakes were found guilty but insane and sent to Broadmoor.^40^
31. Ainsley, ‘Some Mysterious Agency’, 45.
32. Tosh, *Man's Place, *p. 82 and* Manliness and Masculinities in Nineteenth-Century Britain* (Harlow: Pearson Education Limited, 2006), p. 37.
35. BRO, Park's case file, D/H14/D2/2/1/391; Hallowell's case file, D/H14/D2/2/1/905.
36. The Bastardy Act (1845) and the Bastardy Laws Amendment Act (1873) made it legal for the father of a bastard child to be summoned before two justices where he might be ordered to pay a sum for the child's maintenance.
37. ‘Frightful Tragedy: Three Children Murdered’, *Cheshire Observer*, 22 May, 1886, p. 7.
38. W. D. Morrison, *Crime and its Causes* (London: Swan Sonnenschein, 1891), pp. 82–83.
39. ‘A Letter of Despair’, *Lloyd's Weekly Newspaper*, 6 July 1890; *OBPO* (30 September 2012), July 1890, trial of RICHARD ARTHUR OAKES (59) AMY OAKES (46) (T18900728-587).
40. In *The Massacre of the Innocents: Infanticide in Britain 1800*–*1939* (London: Routledge, 1986), Lionel Rose concludes poverty was synonymous with the neglect of children. But cases such as these suggest that some poor families did form emotional bonds with their offspring, thus supporting Julie-Marie Strange's argument that ‘the dynamics of interpersonal relationships were more ambiguous than has previously been allowed’. *Death, Grief and Poverty*, p. 233.


These cases – and the fact the judiciary declared these crimes acts of insanity – indicate the association of economic failure with personal failure in nineteenth-century cultures of masculinity. Such instances suggest, I would argue, that the desire to provide for one's family was not purely a bourgeois construct of masculinity, but a paternal impulse shared by working-class men.^41^ Melissa Gregory's analysis of representations of male infanticide in *The Times* during the early nineteenth century draws attention to the prevalent image of working-class fathers who had internalized their role as providers to such an extent that failure to meet that expectation left child-murder as an alternative to starvation. Gregory concludes that these reports ‘suggested that child-murder was the only way for him to obliterate the pain he felt at his psychological emasculation’.^42^ Such depictions continued to be common throughout the nineteenth century and the expectation a man would provide for his child was not lost on middle-class juries and judges, who sympathized with the struggles these working-class men faced. This emphasis in the courtroom and the press on a man's failure to secure employment or to provide for his child shifted attention from the murdered child to the father. In their studies of infanticidal women Ann Higginbotham and Aeron Hunt suggest that journalistic and judicial attributions of infanticide to illegitimacy and insanity deflected attention from the actual cause of the crime, poverty.^43^ Poverty was harder to ignore in the case of paternal child-murderers as defendants, such as Oakes, sometimes referred to it themselves. The press, however, still managed to avoid the issue by casting defendants as insane.^44^ Thus a man's failure to provide for his family overshadowed the murder of his child and turned him into an object of sympathy, whilst simultaneously emasculating him.41. Davidoff and Hall, *Family Fortunes*, p. 334.
42. Gregory, ‘Most Revolting Murder’, in *Writing British Infanticide*, ed. by Jennifer Thorn, p. 79.
43. Ann Higginbotham, ‘“Sin Of The Age”: Infanticide and Illegitimacy in Victorian London’, *Victorian Studies*, 32.3 (1989), 319–37; Aeron Hunt, ‘Calculations and Concealments: Infanticide in Mid-Nineteenth Century Britain’, *Victorian Literature and Culture*, 34 (2006), 71–94.
44. Gregory, ‘Most Revolting Murder’, in *Writing British Infanticide*, ed. by Jennifer Thorn, p. 78.


In was not just poverty that prevented men providing for their families; drunkenness was also a problem. In 1897 physician George Wilson wrote ‘drunkenness is on the way to mental death’ and in their 1905 report, the Commissioners in Lunacy stated that alcohol was ‘brain poison’.^45^ The detrimental effect of intemperance on the mental faculties had long been agreed upon, not only by the medical profession, but also by the Temperance Movement which spread the word that drinking degraded men ‘below the level of very brutes’.^46^ Martin Wiener indicates that the Victorian crusade against drink meant drunkenness received less tolerance from judges than it had done previously, especially in cases of domestic violence.^47^ When linked to insanity, however, drunkenness was often an aspect of the defence in child-murder cases.45. G. R. Wilson, *Drunkenness* (London: Swan Sonnenschein & Co, Ltd, 1897), p. 53; Lunacy Commissioners quoted in R. Jones, ‘Alcohol and National Deterioration’, in *The Drink Problem*, ed. by T. N. Kelynack (London: Methuen & Co, 1907), pp. 229–39 (p. 237).
46. Samuel Jarrold, ‘An Earnest Appeal to the Working-Men of England, against the Use of Intoxicating Drinks and Tobacco’, *Norwich Cheap Tracts* (Norwich: S. Jarrold, 1860), p. 4.
47. Martin Wiener, *Men of Blood: Violence, Manliness, and Criminal Justice in Victorian England* (Cambridge: Cambridge University Press, 2004), p. 276.


Alienists and physicians echoed the animalistic imagery of temperance literature. L. Forbes Winslow wrote that the drunken man was reduced ‘to the level of the beast’ and described his ‘brutalizing habits’.^48^ In *The Drink Question: Its Social and Medical Aspects* (1889), a work on temperance aimed at the lay population, Kate Mitchell declared ‘no four-footed animal is capable of showing such degraded tendencies, and such an utter want of decency, or committing such unnameable offences as man in a state of intoxication’. It was during this time that ‘the passions ride triumphant over reason’ and crimes were committed.^49^ As if to prove this point, Joseph England ‘cut his child's throat (nearly severing its head) with a table knife, in a fit of passion aggravated by hard drinking’ and the ‘maniacal attack’ which led George Lockin to murder his two children was ‘brought on by drink’.^50^ A single dose of alcohol could disorder the intellect and pervert the moral sentiments, meaning even the casual drinker was liable to ‘become perverted into the fiercest of monsters’ whereby he ‘bursts into rage, seizes the readiest weapon […] is furious and savage, strikes or stabs with double violence’.^51^ To some alienists drunkenness was more than unrestricted impulses. In 1897 George Wilson wrote:

Intoxication to the ordinary observer, is loss of self-control; to the physician, it is the physiological effect of alcohol on the brain. Usually, drunkenness is merely regarded as a vicious habit; scientifically, it is a reduction of mental capacity due to deterioration of the brain tissue.^52^
52. Wilson, *The Pathology of Drunkenness*, pp. 1–2.

In line with this scientific approach, physicians established different forms of alcohol-induced mental disease. This included *delirium tremens*, a form of temporary insanity classified by T. Sutton in 1813, which by 1897 was considered ‘the most impressive and dramatic of the alcoholic neuroses’.^53^ Henry Seyman murdered his son following an attack of *delirium tremens* and was not only represented as diseased but also emasculated by this condition. At his trial Seyman's neighbours testified that they no longer witnessed the once loving father playing with his son, nor had he been going to work.^54^ Physicians and social commentators made distinctions between the normal man and the drunkard. To the physician the intemperate madman was, as Charles Wilson wrote, ‘thoroughly unmanned’, for the incapacity for bodily and mental exertion led to business failure and broken promises.^55^ Insanity removed a man's capacity to be independent and, as in Seyman's case, to work, provide for his family and thus be a good father. In his lecture ‘Courtship & Marriage; or, Special Hints to the Single and Married’ the phrenologist, Reverend John William Taylor warned his readers that intoxicated men transformed from kind and affectionate fathers into cruel tyrants, who make the lives of their wives and children miserable.^56^ Drinking removed men from the homes they were supposed to create and maintain. In *Happy Homes*, J. W. Kirton dedicated a chapter to ‘The Public House, The Rival to Home’ and advised working-class men to spend their money on their home and families rather than on alcohol.^57^
48. L. Forbes Winslow, *On Uncontrollable Drunkenness as a Form of Mental Disorder, With the Only Possible Means of Legally Dealing with Such Cases* (London: Henderson & Spalding Ltd. 1892), pp. 12, 26.
49. Kate Mitchell, *The Drink Question: Its Social and Medical Aspects* (London: Swann Sonnenschein & Co, 1889), p. 200.
50. BRO, England case file, D/H14/D2/2/1/881; Lockin case file, D/H14/D2/2/1/953.
51. Charles Wilson, *The Pathology of Drunkenness: A View of the Operation of Ardent Spirits in the Production of Disease; Founded on Original Observation, and Research* (Edinburgh: Adam and Charles, 1855), p. 127.
53. Wilson, *The Pathology of Drunkenness*, pp. 48–49.
54. ‘The Sheffield Murder’, *Bradford Observer*, 1 April 1869, 7.
55. Wilson, *The Pathology of Drunkenness*, p. 60.
56. John William Taylor, *Love, Courtship, and Marriage: How to Read Character by the Walking, Hand-Shaking, etc, Four Lectures* (London: L. N. Fowler, 1891), p. 52.
57. Kirton, *Happy Homes*, pp. 157–58. For the crusade against drink see Brian Harrison, *Drink and the Victorians: The Temperance Question in England, 1815*–*1872* (Keele: Keele University Press, 1971); Lilian Lewis Shiman, *Crusade Against Drink in Victorian England* (Basingstoke: Macmillan, 1986).


## III. Broadmoor

Regardless of the cause of insanity, the case files suggest that murderous fathers were depressed, regretful and introverted. It was the job of the medical officers at Broadmoor to restore them to the confident, industrious and caring men they once were. Although home to criminal lunatics, Broadmoor resembled many other asylums built in the Victorian period and epitomized the Victorian obsession with moral management, which recognized patients' individual, social and occupational needs.^58^ Patients' insanity was to be remedied through friendly associations and purposeful activities such as reading, exercise, cricket, croquet and chess. Male patients were expected to work in the asylum and were employed at the shoemaker's shop or the tailors, on the asylum farm, in the gardens or as bricklayers or carpenters.^59^ Patients had to display industriousness in Broadmoor before any reference to their potential release was made; they had to show that if they were discharged they would be able to gain and maintain employment in order to provide for themselves and their families. Thus Broadmoor promoted those attributes, including temperance and industriousness, which had previously identified each of the patients examined here as good fathers.58. Deborah Weiner, ‘“This Coy and Secluded Dwelling”: Broadmoor Asylum for the Criminally Insane’, in *Madness, Architecture and the Built Environment*, ed. by Leslie Topp, James E. Moran, and Jonathan Andrews (New York: Routledge, 2007), pp. 137–148.
59. John Meyer, *Reports of the Superintendent and Chaplain of Broadmoor Criminal Lunatic Asylum for the Year 1866* (London: Eyre and Spottiswoode, 1867), p. 6; William Orange, *Reports of the Superintendent and Chaplain of Broadmoor Criminal Lunatic Asylum for the Year 1870* (London: Eyre and Spottiswoode, 1871), p. 10.


The medical reports in patient case files show that when they were committed to Broadmoor, the causes of insanity attributed to paternal child-murderers generally corresponded with what had been argued at their trials and revolved around poverty, domestic troubles and intemperance. When committed, the majority of paternal child-murderers were downcast, regretful and melancholic. Richard Bromley was sullen and unsettled, and preferred ‘a good rest’ to work in the bake house; George Wilson heard voices which caused him ‘nothing but misery and torment’.^60^ Alfred Bligh was more troublesome. He was verbally aggressive, refused to get out of bed and would not work. This led Medical Superintendent Dr. David Nicolson to report to the Secretary of State that although Bligh was ‘well and strong physically’, he ‘has steadily declined to work at any industrial occupation and is of a dissatisfied and grumbling disposition – no doubt natural to him’. Nicolson feared other patients would mimic Bligh's disruptive behaviour and described him as ‘a bad example to others for a man of intelligence’.^61^ He was condemned as lazy, marked as unindustrious, and was refused conditional discharge. Bligh eventually settled down and was conditionally discharged on 2 February 1897 to the care of his sister and brother-in-law.^62^ Bromley and Wilson worked and improved mentally and were discharged; Bromley to his mother and Wilson to his wife. Joseph Wood and Robert Hallowell were also eventually discharged after they too settled down and learnt a trade.^63^
60. BRO, Memorandum, D/H14/D2/2/1/1565; Schedule A, D/H14/D2/2/1/811.
61. BRO, Medical Report, D/H14/D2/2/1/1284/29.
62. BRO, Warrant for Conditional Discharge, D/H14/D2/2/1/1284/37.
63. BRO, Medical Report, D/H14/D2/2/1/1479; Medical Report, D/H14/D2/2/1/905/56.


Obtaining discharge was more complex than proving one's ability to work and it was particularly hard for patients with a history of intemperance. In 1898, three years after his committal, Robert Jones was considered sane and the question of his discharge was raised. During his time in Broadmoor Jones had been industrious, but Medical Superintendent Brayn erred on the side of caution. He warned the Secretary of State that ‘although [Jones] conducts himself satisfactorily under asylum supervision’ his history of intemperance ‘shows that he is of unstable mind’ and he ‘would be liable to relapse if exposed to any worry or anxiety’.^64^ Similar caution was taken in the case of Henry Seyman. A medical report from 1875 shows that Seyman ‘conducted himself well and has worked steadily in the garden’, he was also a ‘trusted performer in the asylum band’, learned to read and write and, for the eight months prior to July 1875, he ‘voluntarily abstained from [the] beer’ which was given to patients at dinner.^65^ In spite of his sanity and good behaviour, caution was advised because his ‘insanity and his crime were caused by intemperance’ and thus ‘[s]imilar events might at any future time ensue should he again give way to drinking habits’.^66^ Broadmoor's Medical Superintendent and the Home Office wanted to be certain that there was minimal risk of patients returning to drink and to ensure that, if released, they were discharged into the care of relatives who would enforce temperance. Seyman's ability to control his temperance, for example, was dependent on ‘the most stringent watchfulness and care on the part of his friends’.^67^ He was conditionally discharged in August 1877 to the care of his brother.^68^ By the time Jones was released into the care of the Salvation Army in 1902, the insanity which followed drinking was considered a ‘real burden to the state’. Patients were discharged into a society in which alcohol was increasingly linked to concerns of racial fitness and national efficiency.^69^ Not only did the friends and relatives of patients have to accept an added ‘Drink Clause’ to the terms of conditional discharge, but patients also had to take ‘The Pledge’ and sign the following statement:

I hereby solemnly promise that, in the event of my conditional discharge from Broadmoor Asylum being sanctioned, I will abstain from all intoxicating drink; and I fully and clearly understand that such discharge will be liable to be revoked at any moment if I fail to keep to the above promise.^70^
70. BRO, ‘The Pledge’, D/H14/D2/2/1/881.

If these promises were broken, the warrant of conditional discharge was revoked and the patient returned to Broadmoor.64. BRO, Medical Report, August 1898, D/H14/D2/2/1/1680.
65. BRO, Medical Report, D/H14/D2/2/1/731/16.
66. BRO, Medical Report, D/H14/D2/2/1/731/12.
67. BRO, Medical Report, D/H14/D2/2/1/731/16.
68. BRO, Warrant for Conditional Discharge, D/H14/D2/2/1/731/21; Letter, D/H14/D2/2/1/731/18.
69. BRO, Warrant for Conditional Discharge, D/H14/D2/2/1/1680; A. Newsholme, ‘Alcohol and Public Health’, in *Drink Problem*, ed. by T. N. Kelynack, pp. 122–51.


The period between patients' arrival and discharge varied. The admission registers for Broadmoor do not list the exact crimes of all the patients committed, but an approximation of the average confinement of child murderers can be established using details of the stays of 85 male child-murderers committed between 1868 and 1900 and 191 female child-murderers committed between 1871 and 1900 recorded in the registers. Those paternal child-murderers who were conditionally discharged spent an average of eleven years in Broadmoor, whilst their female counterparts spent an average of ten years in the asylum. Maternal child-murderers were 1.5 times more likely to be discharged than paternal child-murderers (Table 1). In his study of the discharge of infanticidal women from Broadmoor and Perth asylum, Jonathan Andrews shows that once some women had passed childbearing age and the risk of puerperal insanity they were no longer deemed a threat to society and were discharged.^71^ There was no similar way to assess the threat paternal child-murderers posed which may explain their lower discharge rate in comparison to maternal child-murderers. The discharge figures for the overall Broadmoor population between 1863 and 1900, though, indicate that women were three times more likely to be discharged than men (Table 2). This suggests that sex was less influential in determining the release of female infanticidal patients than in other criminal lunacy cases. Both men and women who killed their children had a better chance of release than the average Broadmoor patient of the same gender, but having murdered one's own child appears to have made more difference to a man's chance of release than to a woman's: paternal child-murderers were 3.1 times more likely to be discharged than the average male, whilst maternal child-murderers were 1.7 times more likely to be discharged than the average female patient. A patient's release was of course dependent upon factors other than sex or crime: their mental and physical wellbeing whilst in the asylum and the availability of friends or relatives willing and able to care for patients upon their discharge were also taken into consideration by the medical officers and by the Home Office.71. Andrews, ‘The Boundaries of her Majesty's Pleasure’, in *Infanticide*, ed. by Jackson, p. 247.


**Table:** Table 1 Duration child-murderers stayed in Broadmoor and mode of release, 1868–1900.

	Non-paternal child-murderers (21)	Paternal child-murderers (58)	Men who killed their wife and child (5)	Man who killed wife and grandchild (1)	Non-maternal child-murderers (5)	Maternal child-murderers (186)
% discharged from Broadmoor	19	28	20	–	0	42
Mean time before discharge (years)	10	11	20	–	–	10
% transferred to another asylum	5	22	40	–	40	15
Mean time before transfer (years)	15	26	11	–	19	21
% who committed suicide	5	2	–	–	–	1
Mean Time before suicide (years)	28	1	–	–	–	4
% who died in Broadmoor	71	45	40	100	40	41
Mean time before death (years)	20	22	19	5	22	28
% transferred to Prison	–	–	–	–	20	1
Mean time before transfer (years)	–	–	–	–	2	1
Unknown	–	–	–	–	–	1

**Table:** Table 2 Means by which all patients were discharged from Broadmoor, 1863–1900.

	Women	Men
% Absolutely Discharged (sane)	2	2
% Conditionally Discharged	23	7
% Died	36	40
% Escaped	–	–
% Not Stated	5	5
% Removed	1	–
% Suicide	–	1
% Transferred to an Asylum	30	38
% Transferred to Prison	2	6

Fathers who had been kind, attentive, hardworking, and temperate prior to murdering their children tended to be found insane when tried for their murder. Once in Broadmoor, it was these same positive characteristics echoing nineteenth-century descriptions of ideal fatherhood that helped determine their discharge from the asylum. Comparing the representation of insane fathers with that two other groups of men who murdered children - childless men and convicted fathers – makes clear just how important nineteenth-century beliefs about fatherhood were in the treatment of child-murder cases.

## IV. Male child-murderers in the late-Victorian press and *Old Bailey Proceedings Online*

Research by A. J. Hammerton, Ginger Frost and Martin Wiener indicates that Victorian judges attempted to improve working-class behaviour through the courts by condemning violent behaviour as unmanly.^72^ My research on male child-murderers supports this broad conclusion. The working and middle classes were intolerant not only of the domestic abuse of women, as scholars have shown, but also that inflicted on children. An examination of press reports and *Old Bailey Proceedings Online* (OBPO) shows that the nature of the crimes committed by childless men and convicted fathers, their motivations for the crime, their previous character, and demeanour in the courtroom were all subject to scrutiny. In condemning the behaviour of these men the press, judges, and juries helped to define appropriate male behaviour, including what made a good father.72. A. James Hammerton, *Cruelty and Companionship: Conflict in Nineteenth-Century Married Life* (London: Routledge, 1992); Ginger Frost, *Living in Sin: Cohabiting as Husband and Wife in Nineteenth-Century England* (Manchester: Manchester University Press, 2008); Wiener, *Men of Blood*, pp. 29–32.


Press representations of child murder differed according to who committed the crime: insane paternal child-murderers tended to be described as pensive and quiet, whereas childless men were seen as savage beasts fuelled by passion. On 29 November 1895, Robert Jones was tried for the murder of Robert Edward Jones, his two-and-a-half-year-old son. He was found insane. On the morning of the crime, Jones got out of bed when one of his three children sleeping in the next room began to cry and, according to the *Huddersfield Daily Chronicle*, returned soon after ‘to horrify his wife with the cool statement, “I have killed Robert”’.^73^ The principal witness was Jones's six-year-old daughter who shared a bedroom with her younger brother and sister. She told the court:

On Saturday morning I woke up. I heard my brother make a noise in his throat as if he was going to be sick. I then saw some blood about him. He was lying at the side of the bed. His head was hanging down, and my father was stooping over him. […] My father went out of the room after that, and left my brother at the foot of the bed. I said, ‘Father, what are you doing’. He did not answer.^74^
74. ‘Another Liverpool Murder’, *Huddersfield Daily Chronicle.*

Jones's quiet, calm completion of the crime was a familiar scene. After Henry Seyman murdered his child, his neighbours heard him calmly say to his wife ‘Go and look at Harry; he is dead’.^75^ Neither Frederick Crawley nor Richard Hammett spoke or moved when discovered with their dead children by their wives and Alfred Bligh convinced his housekeeper to go to the circus before murdering his children at home, alone.^76^ This differed from the crimes of childless men who committed the act in public. John Jordan, Ernest Travers and Frank Brearley, for example, all murdered young children outside; Travers and Brearley did so in front of witnesses. The acts of childless men were reportedly violent and brutal. Various newspapers reported that Hugh Hagan, who murdered the illegitimate child of his cousin's wife, visited his cousin one afternoon and when the child began to cry ‘he immediately burst into a rage, snatched the child from its brother's arms, opened the bed-room door, and “clashed” it upon the bed’.^77^ And Frank Brearley was depicted by the *Derby Mercury* as abnormally strong: although ‘a man and woman had endeavoured to prevent him from murdering the girl […] he proved too strong for them and made a violent attack’. 78 The brutal acts of these men immediately placed them outside the bounds of acceptable masculine behaviour. They were condemned in the press, in the courtroom, and by the public. Travers's case caused such controversy that the police had to take precautions against hostile demonstrations outside the courtroom and his appearance at the pre-trial inquest was met with ‘hooting and groans’ from the crowd outside.^79^
73. ‘Another Liverpool Murder: A Father Kills His Infant Son’, *Huddersfield Daily Chronicle*, 19 August 1895, 3.
75. ‘Awful Tragedy Near Euston Square – Double Murder and Attempted Suicide’, *Illustrated Police News*, 24 September 1881; ‘Shocking Murder’, *Reynold's Newspaper*, 21 March 1869; *Liverpool Mercury*, 24 February 1871.
76. ‘Triple Murder and Attempted Suicide by a Policeman’, *Berrow's Worcester Journal*, 22 May 1884, 2.
77. *Pall Mall Gazette*, 24 February 1873.
78. ‘The Burton Murder’, *Derby Mercury*, 6 August 1890.
79. ‘Sensational Evidence in Court’, *Illustrated Police News*, 20 March 1897.


According to press and medical reports, the motivations of non-paternal child murderers stemmed from deep-seated hostility. John Jordan, for instance, was reported in the press to have murdered out of revenge. Hugh Hagan was initially sentenced to death but was reprieved after two alienists, including Broadmoor's Dr. William Orange, examined him and concluded that ‘intense hatred’ had caused his insanity and thus his crime.^80^ Compared to the pleas of poverty or grief made in the trials of fathers who were eventually found insane for the murder of their child, these were unlikely to induce any sympathy from the press or in the courtroom.80. ‘Barbarous Murder’, *Lloyd's Weekly Newspaper*, 12 May 1872; BRO, Schedule A, D/H14/D2/2/1/779/4.


Fathers convicted for the murder of their child were reportedly driven by similar motives to childless men. The love and devotion insane paternal child-murderers had shown to their children prior to murdering them led some defence counsels' to brand the crime motiveless.^81^ The same was not argued during the trials of convicted fathers. An analysis of *OBPO* indicates that a number of fathers convicted for the murder of their child had previously abused and neglected their wives and children. Walter Marsh's neighbour ‘begged him […] to take care of his family’, while James Cole was depicted as a violent habitual criminal who abused his wife and both John Richard Jefferey's and Clarence Longman's mothers-in-law told the courtroom at their trials that the men had verbally abused their children.^82^ Longman, for example, ‘did not seem to treat the child very kindly’ he called ‘the child a little *sod* more than once. I never saw him hold it in his arms and appear quite pleased with it’. Longman also refused to work.^83^ Whereas hatred and a general disregard for the wellbeing of their child were perceived to motivate these fathers, others were seen to be led by deceit. On 25 August 1879, James Dilley was executed for the murder of his three-week old daughter. Dilley had a wife and family in Bedfordshire and a mistress, a domestic servant named Mary Rainbow, with whom he had fathered the child, in London. Both Dilley and Rainbow were charged with the murder, but Rainbow was reprieved after the Home Secretary accepted that Dilley had coerced her into helping him commit the crime. At the trial, Dilley was portrayed by Rainbow's defence as a ‘dastardly and cruel’ man whose seduction had ruined Rainbow's reputation. His adultery and the consequent neglect of his wife and children, along with his alleged coercion of Rainbow to murder their illegitimate child, did not invoke sympathy either from the press or in the courtroom. According to the judge, Dilley's was a deliberate act of murder, motivated by his wish ‘to prevent the fact of the birth of the woman becoming known to his wife, and to escape the burden of having to support it’.^84^ And, echoing press reactions to childless murderers, the judge told Dilley during sentencing that his crime was ‘a base, a cruel, and barbarous murder’.^85^ Convicted fathers thus feature in the Old Bailey trial proceedings as men deficient in what was understood as the innate desire to provide for and protect their children.81. Some alienists and laymen also considered a lack of motive evidence of insanity. Roger Smith, *Trial by Medicine: Insanity and Responsibility in Victorian Trials* (Edinburgh: Edinburgh University Press, 1981), pp. 121–22.
82. *OBPO* (02 February 2012), October 1883, trial of JAMES COLE (37) (t18831015-964); *OBPO* (27 June 2012), June 1866, trial of WALTER MARSH, (47) (t18660611-565); *OBPO* (27 June 2012), September 1866, trial of JOHN RICHARD JEFFEREY, (31) (t18660917-757).
83. *OBPO* (02 February 2012), November 1889, trial of CLARENCE HENRY LONGMAN (19) (t18891118-53).
84. *OBPO* (02 February 2012), August 1879, trial of JAMES DILLEY (41) MARY RAINBOW (28) (t18790805-698).
85. ‘Central Criminal Court’, *Morning Post*, 25 November 1889, 3; ‘The Hornsey Murder: Sentence’, *York Herald*, 11 August 1879, 8.


Paternal infanticide was considered a ‘sad tragedy’ by the press and headlines such as ‘A Father's Terrible Crime’ and ‘A Father's Awful Crime’ reinforced this representation.^86^ Insane paternal child-murderers were depicted in the press as remorseful, withered wrecks. The *Pall Mall Gazette* reported that when Robert Jones left court ‘five policemen had to support the wretched man, who was in a state of utter collapse, and pathetically enquired whether his little son was dead’.^87^ William Kemp was ‘so prostrated that a medical man remained with him in the dock, and [he] was kept in a state of consciousness by the repeated application of stimulants’.^88^ Kemp also ‘sat with his face buried in his hands’ and ‘occasionally shed tears’, as did Richard Hammett and Richard Oakes.^89^ Frederick Crawley ‘cried piteously’ during his trial and Henry Seyman ‘covered his face with a handkerchief, and at times cried very bitterly’.^90^ Childless men and convicted fathers, by contrast, featured as ‘culprits’ and murderers under headlines such as ‘Shocking Child Murder’, ‘Barbarous Murder’, and ‘Horrible Murder’.^91^ Press representations of childless men during their trials differed from the emotional father. Few references were made to Hugh Hagan's demeanour during his trial other than his propensity for passionate violence, John Jordan was ‘cool and collected’, and Ernest Travers was ‘indifferent’ to the frightful evidence produced against him.^92^
86. ‘A Father's Awful Crime’, *Glasgow Herald*, 19 August 1895; ‘A Father's Terrible Crime’, *Pall Mall Gazette*, 19 August 1895.
87. ‘A Father's Terrible Crime’, *Pall Mall Gazette*.
88. ‘Murder by a Father’, *Morning Post*, 3 December 1868, 3.
89. ‘The Shocking Tragedy Near Rugby’, *Birmingham Daily Post*, 8 July 1868; ‘Murder of Two Children’, *Lancaster and General Advertiser for Lancaster, Westmorland and Yorkshire*, 10 December 1881.
90. ‘Shocking Case of Murder Mania in Liverpool’, *Cheshire Observer*, 5 February 1871, 5; ‘The Murder in Lansdowne Road’, *Sheffield and Rotherham Independent*, 27 March 1869, 11.
91. ‘Shocking Murder of a Boy: Culprit Caught Red-Handed’, *Aberdeen Weekly Journal*, 10 March 1897; ‘Barbarous Murder’, *Lloyd's Weekly Newspaper*, 12 May 1872; ‘A Father's Terrible Crime’, *Pall Mall Gazette*, 19 August 1895.
92. ‘Barbarous Murder’, *Lloyd's Weekly Newspaper*; ‘Sensational Evidence in Court’, *Illustrated Police News*.


Newspaper reports and trial transcripts support the argument that paternal infanticide was commonly construed as so counter to ‘natural’ affections that it was clear grounds for a plea of insanity. Childless men who killed children were vilified in the press. A father's murder of his child was, by contrast, judged according to the perception of motive and previous performance of fatherly duty. Comparison of press representations of insane paternal child-murderers and convicted fathers suggests that the press endorsed a specific paternal identity, with protection and provision, love, temperance and industriousness at its core. The court provided legal reinforcement of such notions: judges and juries condemned the behaviour of convicted fathers who had fallen short of such ideals and tended to see fathers who had previously conformed to notions of appropriate fatherhood as insane.

When childless men were found insane, which sometimes happened following their conviction, the negative representations of them found in the press and in the courtroom were echoed in Broadmoor. Ernest Travers was described by Medical Superintendent Dr. Richard Brayn as ‘a most plausible, cunning, unscrupulous man, in whom neither the staff nor his fellow patients place any trust or confidence’.^93^ The depictions of John Jordan, Hugh Hagan and Frank Brearley in Broadmoor were similar: they were all represented as wild, dangerous, and threatening; a stark contrast to the depiction of insane fathers who were melancholic and downcast, although well behaved and industrious.^94^ Paternal child-murderers were 1.5 times more likely to be discharged from Broadmoor than childless men who killed children. Moreover, 71% of non-paternal child murderers committed to the asylum died in confinement in comparison to 45% of paternal child-murderers (Table 1).^95^ The previous behaviour of insane paternal child-murderers and their demeanour within Broadmoor may explain why they were more likely to be conditionally discharged or transferred to another asylum than childless men.93. BRO, Letter to the Commissioners, D/H14/D2/2/1/1738.
94. BRO, Brearley case file, D/H14/D2/2/1/1492; Hagan case file, D/H14/D2/2/1/779; Jordan case file, D/H14/D2/2/1/754; Travers case file, D/H14/D2/2/1/1738.
95. An examination of the numbers contained in Tables 1 and 2 suggests that childless murderers were 2 times more likely to be discharged from Broadmoor than men in the general asylum population. Paternal child-murderers however, were 3 times more likely to be discharged than the average male patient.


The trials and verdicts discussed in this article indicate the widespread support and enforcement of a specific model of fatherhood in the Victorian period: loving, caring, temperate and playful, with the desire and drive to financially support and protect one's child. The horror witnesses expressed at the way convicted fathers had previously addressed their children and a neighbour's concern that the intemperate Henry Seyman had stopped playing with his child, indicates that members of the working classes expected fathers to adhere to this model of fatherhood. Insane paternal child-murderers were represented as having accepted what it meant to be a good father and as having committed the crime because of their perceived or real failings in this respect. It was not portrayed as an act borne out of the desire to conceal deceit, nor was it committed in anger or out of revenge, as was the case for convicted child murderers. Rather, it was a selfless act carried out in a state of depression, alone and silently: a final act of mercy for the child they feared doomed to destitution, failure or murder.

## V. Conclusion

Meg Arnot has argued that, in comparison to men, infanticidal women were rarely convicted and hanged in nineteenth-century Britain.^96^ But some women were convicted and an examination of these cases highlights some interesting parallels between cases of male and female infanticide. In 1870 two baby-farmers, Margaret Waters and Sarah Ellis, were tried at the Old Bailey for murder, manslaughter, conspiracy and obtaining money under false pretences after one child in their care died and others were found dirty and emaciated. Waters, a childless widow whose motivation for caring for children was considered financial, was convicted and hanged. Ellis, a married (but separated) mother of one, was convicted of the lesser charge of obtaining money under false pretences and sentenced to eighteen months hard labour. Arnot argues that this case ‘emphasised the privilege given to natural mothers’ and suggested that because Ellis was ‘fulfilling the duty of motherhood’ she was spared the capital conviction.^97^ But a careful study of representations of murderous fathers suggests that attitudes towards paternal child-murder were more similar to those expressed in cases of female infanticide than has been supposed. The evidence suggests that childless men, bad fathers and neglectful husbands, were convicted and sometimes hanged for child murder, but if it could be shown that infanticidal men had previously fulfilled their duties as husbands and fathers, they tended to be considered insane and committed to Broadmoor.96. Meg Arnot, ‘The Murder of Thomas Sandles: Meanings of Mid-Nineteenth-Century Infanticide’, in *Infanticide*, ed. by Mark Jackson, pp. 149–67 (p. 163). There is scholarly consensus that in the nineteenth century women were less likely to be punished for violent crimes than men. See, Barry S. Godfrey, Stephen Farrall, & Susanne Karstedt, ‘Explaining Gendered Sentencing Patterns for Violent Men and Women in the Late-Victorian and Edwardian Period’, *British Journal of Criminology*, 45 (2005), 696–720.
97. Meg Arnot, ‘Infant Death, Childcare and the State: The Baby-Farming Scandal and the First Infant Life Protection Legislation of 1872’, *Continuity and Change*, 9 (1994), 271–311 (p. 279).


